# Case report: Recovery of hallucinations and cognitive impairment after administration of donepezil in a patient with schizophrenia and carbon monoxide poisoning

**DOI:** 10.3389/fpsyt.2022.1071417

**Published:** 2022-11-18

**Authors:** Seon-Hwa Baek, Ju-Wan Kim, Yun Young Lee, Ju-Yong Kim, Sung-Wan Kim, Jae-Min Kim

**Affiliations:** ^1^Departments of Psychiatry, Chonnam National University Medical School, Gwangju, South Korea; ^2^Departments of Radiology, Chonnam National University Medical School, Gwangju, South Korea

**Keywords:** suicide, carbon monoxide, delayed neurological sequelae, donepezil, schizophrenia

## Abstract

**Objectives:**

This report aims to introduce a rare case of a dramatic recovery by donepezil with a patient with schizophrenia who suffered from remaining psychotic symptoms despite proper treatment and had a cognitive impairment by carbon monoxide (CO) poisoning sequelae.

**Case report:**

A 38-year-old male who developed schizophrenia 2 years ago had attempted suicide *via* CO inhalation due to his uncontrolled symptoms. He was hospitalized with delayed neurological sequelae (DNS). Though hyperbaric oxygen therapy (HBOT) was applied 10 times, his cognitive impairment did not recover. Surprisingly, with 5–10 mg donepezil, both cognitive function and the psychotic symptoms of the patient remarkably improved.

**Conclusion:**

This case showed a good response of donepezil for a patient with schizophrenia and CO-induced DNS after ineffective HBOT. Although the mechanism of the phenomenon is unclear, it can be possible reasons that the neuroprotective effect of donepezil and white matter insult by CO poisoning.

## Introduction

The lifetime risk of suicide rate among patients with schizophrenia is approximately 14.1% in the global population, and 26.8% of individuals with schizophrenia attempt suicide (1, 2), and the psychotic symptoms, as well as paranoid delusions and commanding hallucinations, are associated with a high risk of suicide (3).

Carbon monoxide (CO) poisoning by burning charcoal is one of the most popular methods of suicide (4). The symptoms of CO poisoning range from slight headache and anxiety to the most severe manifestations such as urinary/stool incontinence, inability to walk, and cognitive impairment (5). Delayed neurological sequelae (DNS) can be developed according to the duration of CO exposure. It can be defined as severe neuropsychiatric symptoms developed after 2–40 days of lucidity in patients with CO poisoning (6–8). Although hyperbaric oxygen therapy (HBOT) is commonly applied to patients with DNS there are no randomized trials showing improvement of neuropsychological symptoms by HBOT in patients with CO poisoning (9, 10).

Few available treatments are for DNS, despite proposed treatments including steroids, aspirin, cerebral vasodilators, bromocriptine, L-dopa, barbiturates, and dextroamphetamine (9, 11–15). Several case reports recently found that acetylcholinesterase inhibitors (AchEIs) were effective in cognitive impairment (16, 17). Interestingly, we experienced an episode of a patient with schizophrenia and CO-induced DNS who responded well to donepezil after unsuccessful HBOT. This case gives an example as the first report for a patient with schizophrenia using donepezil for DNS.

## Case report

A 38-year-old male diagnosed with schizophrenia is currently in partial remission. He had visited the hospital for 2 years to treat psychotic symptoms including auditory hallucinations and persecutory delusions. These symptoms had developed a week before his first visit to the hospital. He had no family history related to psychiatric or other medical conditions. He worked in a large company after graduating from college. On being diagnosed with schizophrenia, he took medicines: amisulpride 800 mg, procyclidine 5 mg, propranolol 80 mg, and diazepam 2 mg. His psychotic symptoms were improved after medication, while auditory hallucinations remained unchanged. He reported that he found it hard to work because the voices reproached him. After consulting a doctor, he stopped the work for a moment due to aggravated symptoms. Although the doctor recommended him admission for controlling residual hallucinations, he insisted on keeping outpatient clinic treatment.

**Figure 1:**
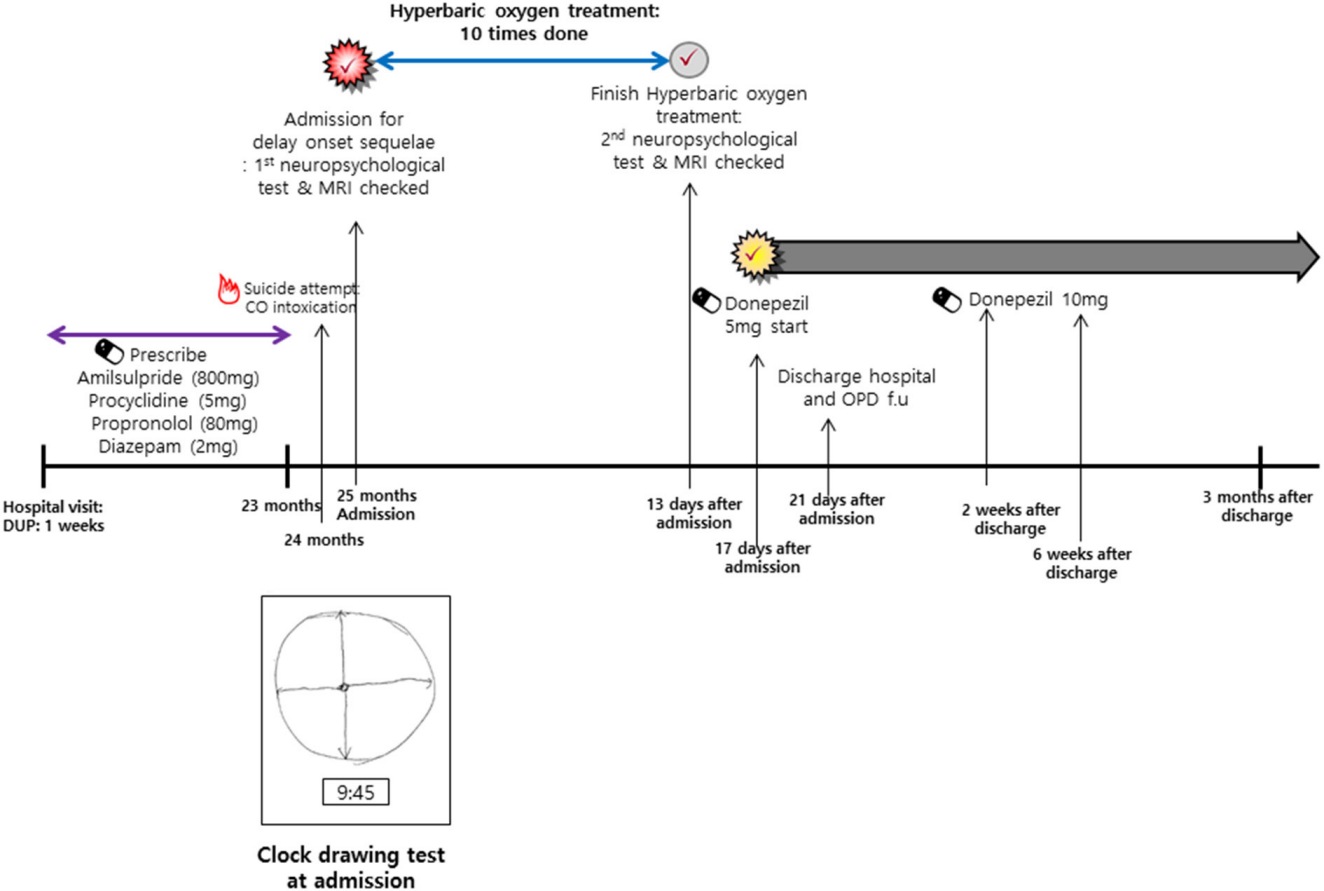
Timeline of clinical course before and after CO intoxication. PANSS, Positive and Negative Syndrome Scale; MINI, Mini-International Neuropsychiatric Interview; CGI-S, Clinical Global Impressions- Severity; CGI-I, Clinical Global Impressions-Improve.

With uncontrolled residual psychotic symptoms, he attempted suicide by inhaling charcoal fumes. He was taken to an emergency room at a hospital and underwent symptomatic care, not HBOT, for CO poisoning and rhabdomyolysis. A month later, severe cognitive dysfunction occurred to him. It was composed of memory decline, gait disturbance, and ataxia at the time of admission. He showed marked deterioration of visuospatial function; he stated that he lost all sense of direction. In a clock-drawing test performed on the day of evaluation, he could not tell the hour hand from the minute hand and could not draw the hour hand (as dictated by a doctor; Figure 1).

**Figure 2:**
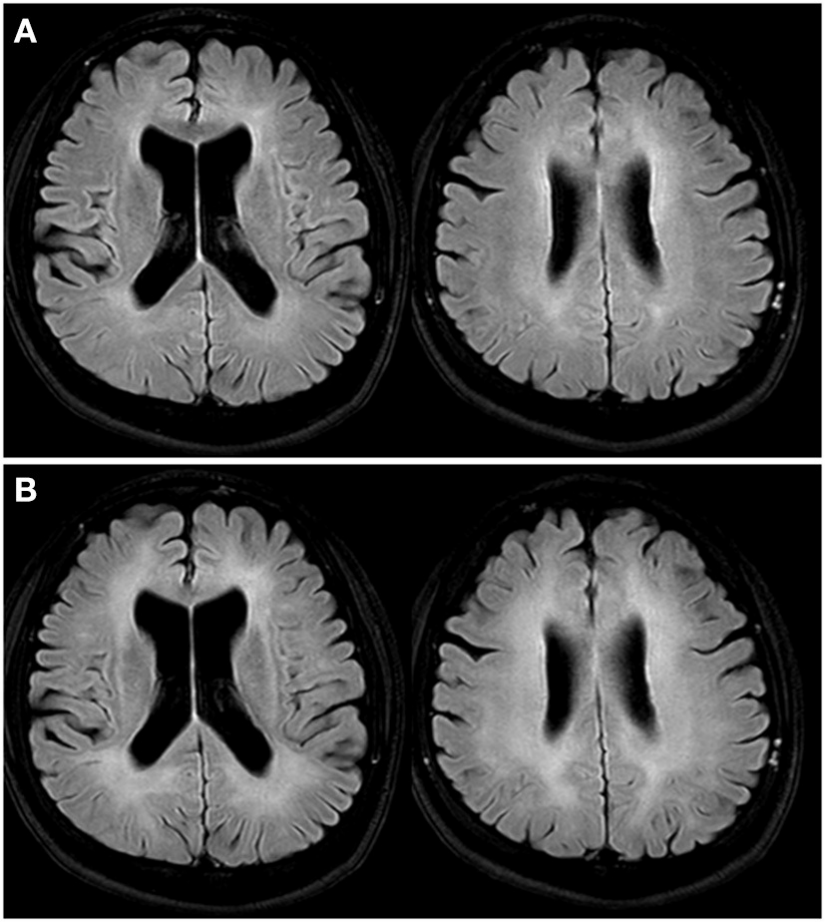
Brain MRI T2 fluid-attenuated inversion recovery image. **(A)** Initially, it shows bilateral symmetric confluent high signal intensities in periventricular white matters of cerebral hemispheres, which are compatible with delayed CO encephalopathy. **(B)** After HBOT shows increases in the extent of confluent high signal intensities in bilateral cerebral periventricular and subcortical white matter. MRI, magnetic resonance imaging; CO, carbon monoxide; HBOT, hyperbaric oxygen therapy.

Due to his deteriorated state, physicians decided to apply HBOT and taper psychotropic medications. Brain magnetic resonance imaging (MRI) and a neuropsychological test were executed to confirm his structural and functional brain conditions before therapy. The tests were repeated after 10 times HBOT. The treatment schedule is shown in Figure 1. Pre-and-post-HBOT data are shown in Table 1. In short, HBOT did not change his brain structure and function (Figure 2 and Table 1).

**Table 1:** Cognitive function profiles before and after hyperbaric oxygen therapy.

	**Before HBOT**	**After HBOT**
**Intellectual functioning test, score (percentile)**		
VCI	74 (4)	55 (0.1)
PRI	55 (0.1)	50 (< 0.1)
WMI	72 (3)	50 (< 0.1)
PSI	50 (< 0.1)	50 (< 0.1)
FSIQ	54 (0.1)	42 (< 0.1)
**Rey-Kim memory test**		
K-BNT, score (%)	51 (41)	2 < 0.1
MQ, score	56	51
**Frontal lobe function**		
EIQ, score	49	51

HBOT, hyperbaric oxygen therapy; VCI, verbal comprehension index; PRI, perceptual reasoning index; WMI, working memory index; PSI, processing speed index; FSIQ, full-scale intelligence quotient; K-BNT, Korean version-Boston Naming Test; MQ, Memory Quotient; EIQ, executive IQ.

We used the scale Positive and Negative Syndrome Scale (PANSS), Clinical Global Impressions-Severity (CGI-S), Clinical Global Impressions-Improvement (CGI-I), and Mini-Mental State Examination (MMSE) to assess the psychotic symptoms and cognitive dysfunction (18–21). Despite continuous treatment, the overall patient's cognitive function and psychiatric status had been unchanged without worsening during the 10 times HBOT sessions. Changes in symptoms through pre-and-post therapy are shown in Table 2. He was somewhat inconsistent and ambiguous when discussing his underlying psychotic symptoms. For getting more information on him, we had a retrospective interview with a caregiver. Regardless of HBOT, mild to moderate psychotic symptoms and very severe depression were found consistently.

**Table 2:** Scores on assessment scales of cognitive function and psychotic symptoms assessment.

	**Baseline** ** (The first visit)**	**24 months** ** (CO intoxication)**	**25 months** ** (Admission)**	**13 days after admission** ** (Finish HBOT)**	**17 days after admission** ** (start donepezil 5 mg)**	**21 days after admission** ** (discharge)**	**2 weeks after discharge** ** (titration donepezil 10 mg)**	**6 weeks after discharge**	**3 months after discharge**
MMSE	28		19	18	19	22	26	30	30
CGI-S			6	6	6	5	3	1	1
CGI-I			4	4	4	3	2	1	1
PANSS	27/14/37/78	16/18/36/70							8/14/29/51

HBOT, hyperbaric oxygen therapy; MMSE, Mini-Mental State Examination; CGI-S, Clinical Global Impressions-Severity; CGI-I, Clinical Global Impressions-Improvement; PANSS, Positive and Negative Syndrome Scale.

We prescribed donepezil 5 mg once daily to him; after 4 days, the MMSE score improved from 19 to 22. He explained that his subjective cognitive decline had improved by ~70% at the time of discharge. He had no difficulty in activities of daily living and gradually improved with gait. Despite the short-term treatment donepezil seemed to be effective. The dose was increased to 10 mg daily to improve cognition further. He could deal with household matters without any other's help. His MMSE score was 30; additionally, he stated that psychotic symptoms had disappeared as increasing the dose of donepezil. Three months after discharge, remaining on donepezil, he is mentally stable and has returned to work. Supplementary Figure 1 shows a change in scores on the Clinician-Rated Dimensions of Psychosis Symptom Severity (Dimensional) scale in the DSM-5 (22), both the psychotic and cognitive symptoms.

## Discussion

We report a case of a patient with schizophrenia in which DNS and functional impairment following CO poisoning improved greatly after a prescription of donepezil hydrochloride, an AchEI. Before donepezil treatment, he had undergone HBOT for 10 sessions which did not improve DNS. His cognitive function increased dramatically on donepezil, such that he has been able to return to work.

Our study has two major clinical implications. First, donepezil may be useful for treating DNS caused by CO poisoning (whereas HBOT was ineffective). The underlying cause of DNS after CO poisoning remains unclear but may be due to diffuse demyelination of the cerebral white matter (23–25). Hippocampal nicotinic acetylcholinergic neurons were dysfunctional in an animal model of DNS induced by CO poisoning (26). Donepezil exerts anti-inflammatory and neuroprotective effects (27–29). However, the effects of donepezil on DNS are not known; randomized controlled trials are needed. Second, before attempting suicide, the patient reported severe auditory hallucinations despite adequate medication. When we interviewed the patient after he had been started on donepezil, his cognitive function was observed to have improved markedly, and all psychiatric symptoms including the auditory hallucinations had disappeared. Earlier case reports found that psychiatric symptoms before CO poisoning, as well as DNS symptoms, persisted in some patients but disappeared in others after starting on donepezil (16, 17, 30). Underlying psychiatric symptoms improved only in patients with mood disorders; they persisted in those with other psychotic symptoms, although cognitive function improved in all patients. Meanwhile, there was one case of schizophrenia with the delusion that responded to donepezil augmented with antipsychotics (31). However, the only use of donepezil to improve psychotic symptoms has been hard to find any reports. Our findings differ; the DNS and psychotic symptoms disappeared completely after we prescribed donepezil without antipsychotics. The disappearance of psychotic symptoms that did not completely respond to earlier (pre-DNS) medications may be explained as follows. First, increasing evidence suggests that the cerebral white matter is involved in the pathophysiology of schizophrenia (32, 33). In this case, white matter abnormalities caused by DNS may have improved the psychiatric symptoms. Second, the neuroprotective effect of donepezil might improve psychiatric symptoms. Third, the suicide attempt *per se* may have exerted a psychological effect, possibly improving the psychotic symptoms. However, further study is warranted. In particular, any future recurrence of psychotic symptoms will be clinically relevant.

Despite the demonstration of a clear effect of donepezil in a patient with schizophrenia and CO-induced DNS this paper has limitations. First, we could not conclude what mechanism is underlying this phenomenon. Second, we could not find similar cases elsewhere. Third, In this case, when the patient came to the emergency room of our hospital, it had been a month since CO intoxication occurred, and acute blood samples, including a percentage of carboxyhemoglobin, could not be obtained. However, delayed encephalopathy of CO intoxication had been confirmed from the brain MRI readings performed at our hospital. Accordingly, it could diagnose through the correlation between the medical history and diagnosis at the time of inhalation and the patient's clinical symptoms and images.

We described a patient with schizophrenia and CO-induced DNS who responded well to donepezil after ineffective HBOT. Further studies are warranted to confirm the long-term efficacy of donepezil for improving cognitive function and psychotic symptoms in patients with schizophrenia, particularly after CO intoxication.

## Data availability statement

The original contributions presented in the study are included in the article/Supplementary material, further inquiries can be directed to the corresponding author/s.

## Ethics statement

Ethical review and approval was not required for the study on human participants in accordance with the local legislation and institutional requirements. The patients/participants provided their written informed consent to participate in this study. Written informed consent was obtained from the individual(s) for the publication of any potentially identifiable images or data included in this article.

## Author contributions

Had full access to all of the data in the study and take responsibility for the data: S-HB, J-WK, and J-MK. Study concept and design: J-WK and S-WK. Drafting of the manuscript: J-WK and J-MK. Critical revision of the manuscript for important intellectual content: J-WK, YYL, J-YK, S-WK, and J-MK. Administrative, technical, or material support: YYL and J-YK. Study supervision: S-WK and J-MK. All authors contributed to the article and approved the submitted version.

## Funding

The study was funded by a grant of National Research Foundation of Korea Grant [NRF-2020M3E5D9080733] and [NRF-2020R1A2C2003472].

## Conflict of interest

The authors declare that the research was conducted in the absence of any commercial or financial relationships that could be construed as a potential conflict of interest.

## Publisher's note

All claims expressed in this article are solely those of the authors and do not necessarily represent those of their affiliated organizations, or those of the publisher, the editors and the reviewers. Any product that may be evaluated in this article, or claim that may be made by its manufacturer, is not guaranteed or endorsed by the publisher.

## Supplementary material

Supplementary Figure S1 Changes in scores on the Clinician-Rated Dimensions of Psychosis Symptom Severity (Dimensional) scale in the DSM-5.
