# Supplementary figures and images for: Case report: Recovery of hallucinations and cognitive impairment after administration of donepezil in a patient with schizophrenia and carbon monoxide poisoning
Source: Front Psychiatry. 2022 Nov 18;13:1071417. doi: 10.3389/fpsyt.2022.1071417 (PMC9715735; doi:10.3389/fpsyt.2022.1071417)

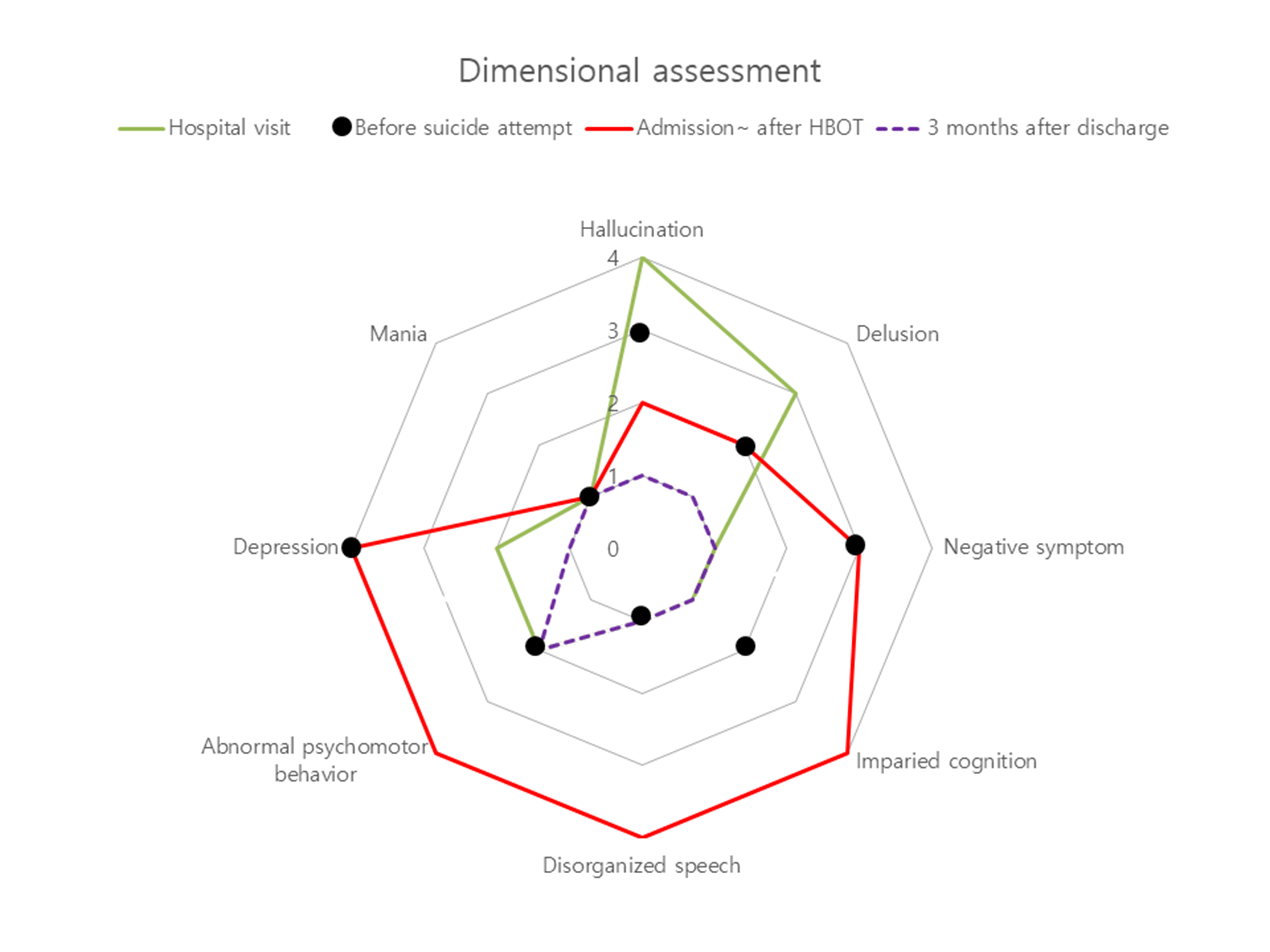

Supplement: Supplementary Figure S1 — Changes in scores on the Clinician-Rated Dimensions of Psychosis Symptom Severity (Dimensional) scale in the DSM-5. [file Image_1.png]
